# Transcatheter Embolization in Congenital Cardiovascular Malformations—Variable Use of Vascular Plugs

**DOI:** 10.1155/2024/4778469

**Published:** 2024-05-29

**Authors:** Jochen Pfeifer, Anas Gheibeh, Peter Fries, Martin Poryo, Axel Rentzsch, Hashim Abdul-Khaliq

**Affiliations:** ^1^ Department of Pediatric Cardiology Saarland University Medical Center, Homburg 66421, Germany; ^2^ Clinic for Diagnostic and Interventional Radiology Saarland University Medical Center, Homburg 66421, Germany

## Abstract

**Objective:** The objective of this study is to evaluate the clinical application and primary outcome of transcatheter embolization using Amplatzer™ Vascular Plug (AVP) Type 2 and Type 4 in different congenital cardiovascular malformations.

**Design:** This is a single-center retrospective observational cohort study.

**Methods:** We analyzed clinical and imaging data of 36 patients retrospectively who received transcatheter embolizations of the following malformations using AVP: systemic-to-pulmonary collateral arteries (SPCA), patent ductus arteriosus (PDA), ventricular septal defects (VSD), and aberrant pulmonary sequestration arteries (PSA). We included all patients treated in our institution from January 2010 to July 2023.

**Results:** In 36 patients (median age 40.0 months, range 0.5 months–42.0 years; 56.8% male), 44 AVPs were implanted in 37 procedures. The target lesions were SPCA in *n* = 15 procedures, PDA in *n* = 9, VSD in *n* = 9, and PSA in *n* = 4. Thirty-four AVP Type 2 and 10 AVP Type 4 were applied, the latter only in SPCA and PSA. SPCA was most common in complex congenital heart disease with univentricular physiology (75.0%). VSD were associated with additional cardiac malformations in 33.3%, PDA were associated with prematurity (55.6%), and all pulmonary sequestrations occurred in scimitar syndrome. Primary total or subtotal occlusion succeeded in *n*38/44 (86.3%). For residual PDA, an additional occluder was implanted in one patient. In one case, pulmonary sequestration had to be treated surgically. One premature infant with PDA closure sustained a relevant obstruction of the left pulmonary artery by the outer AVP disc which required surgical correction 4 months later.

**Conclusion:** Embolization using AVP is a suitable approach for closure of various cardiovascular malformations with a high primary success rate and low complication rate. It should be considered in treatment of different irregular vessel anomalies and in selected VSD.

## 1. Introduction

Irregular vessels and shunts are common manifestations of congenital cardiovascular malformations. Frequently, their closure is clinically indicated. However, due to the diversity of types, localizations, and variability of size and shape of the targeted structures, the selection of a suitable therapeutic approach can be challenging. Apart from surgical repair, transcatheter embolization represents an alternative approach yielding a less invasive yet definite therapy or serving as preparation for a subsequent surgery.

Various mechanical devices are available for endovascular embolization, such as septal occluders, coils, and vascular plugs. Amplatzer™ Vascular Plugs (AVPs) (Abbott Medical, Plymouth, MN, USA) are self-expanding nitinol devices which can be used for a wide range of acquired and congenital diseases [1–7]. Configuration and characteristics of these devices are well described [4, 8]. In the context of congenital abnormal vessels, the AVP Type 2 (AVP-2) and AVP Type 4 (AVP-4) are most frequently applied. The latter qualifies especially for tortuous and small vessels, while AVP-2 is used for large-lumen vessels and various landing zones. In addition, AVP-2 has two stable lateral discs which allow the device to be anchored more securely in shunts than conventional duct occluders. Among other rare manifestations, systemic-to-pulmonary collateral arteries (SPCA), patent ductus arteriosus (PDA), ventricular septal defects (VSD), and pulmonary sequestration arteries (PSA) are congenital malformations suitable for transcatheter closure using AVP. These pathological lesions should be occluded due to various indications as stated in the following.

SPCA typically occur in complex congenital heart defects (CHD), especially univentricular hearts and partial or total cavo-pulmonary connection (TCPC) (Fontan circulation), as well as in premature infants [9, 10]. They are usually derived from the thoracic aorta or its branches, namely, from the subclavian, internal mammary, thyroid, or carotid arteries [11]. Occlusion of SPCA can be necessary as they can cause both volume and pressure overload especially in single ventricle anatomy [12] and can promote deterioration in Fontan circulation [13]. Left-to-right shunt via PDA or VSD may result in cardiac volume overload or pulmonary hypertension [14].

Sequestration of the lung is frequently associated with CHD, especially with scimitar syndrome [15, 16]. Pulmonary sequestration can cause recurring pneumonia or hemoptysis; embolization of PSA may either be the definite treatment or precede surgical resection of the sequestration [17].

The aim of our retrospective study was to analyze the primary success and possible complications using AVP of various types and sizes in embolization of the above-mentioned cardiovascular target lesions.

## 2. Patients and Methods

After approval from the local ethics committee of the Saarland, Saarbruecken, Germany (file number 154/23), this retrospective single-center study was performed at the Saarland University Medical Center, Homburg, Saarland, Germany.

We analyzed all patients with congenital cardiovascular malformations retrospectively, who underwent transcatheter embolization by AVP (Abbott Medical, Plymouth, MN, USA) in our institution from January 2010 to July 2023. In our center, AVPs are used in patients affected by either SPCA, PDA, VSD, or pulmonary sequestration. Concerning these malformations, a total number of 286 embolization procedures were performed during the study period, thereof 100 in SPCA, 150 in PDA, 32 in VSD, and 4 in PSA, respectively, using different types of mechanical devices. Specifically AVPs had been applied in 37/286 (12.9%) procedures, namely, in 15/100 (15%) procedures for SPCA, in 9/150 (6%) for PDA, in 9/32 (28.1%) for VSD, and in 4/4 (100%) for PSA, respectively.

**Figure 1:**
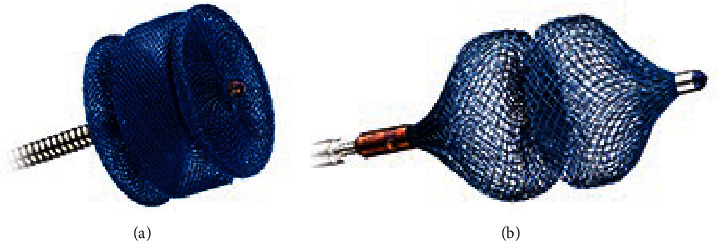
(a) AVP-2. (b) AVP-4. *Source:* (The Amplatzer™ Family of Vascular Plugs), viewed on 15 April 2024.

Occlusion was performed with AVP-2 and AVP-4 of different sizes (Figure 1 shows both AVP types). Either the patients or the parents/guardians had given written informed consent before the procedure.

AVP had been implanted into malformations with the following characteristics:

- SPCA or PSA with long landing zones,
- Tortuous SPCA,
- Tubular and elongated PDA with large diameters,
- Tunnel-like muscular or aneurysmatic perimembranous VSD.

For all procedures, the lesions' diameters were sized in the fluoroscopic images prior to embolization. In order to ensure stable device position and closure as complete as possible, the device has to be oversized in relation to the targeted diameter. Usually, we aimed to oversize 30–50%, as recommended. Furthermore, the selection of the AVP size depends on the length of the landing zone. If this was more than 10 mm, an AVP was chosen over another device because AVPs have a significantly greater constrained length after implantation and will thus cover a longer section. Considering these requirements as well as the lesions' morphology and the available AVP types, a deviation from the recommended oversizing was inevitable in selected cases.

In both VSD and PDA, the AVP has to be anchored on both sides of the lesion. In these cases, only AVP-2 is appropriate due to its trilobar design whereas AVP-4 is not suitable. Instead of other types of occluders, we chose AVP in elongated PDA and tunnel-like muscular or aneurysmatic perimembranous VSD with lengths of more than 6 mm due to the greater length of the AVP especially after constraining.

We assessed the embolization procedures regarding patients' demographics, diagnoses, and localizations of embolization, as well as number and sizes of the AVP, extent of device oversize, primary success, and possible complications of the procedures. The diameters of the target lesions were remeasured in the angiographic records. Successful closure was defined as total or subtotal occlusion (i.e., minimal residual shunt) either at the end of the intervention (fluoroscopically evaluated) or before discharge (evaluated by color Doppler imaging or by computed tomography angiography). Adverse events included vascular injury or rupture, hemorrhage, migration of the device, and obstruction of adjacent or nontarget vessels during the intervention or at any time during follow-up.

Data analysis is purely descriptive. The data is given as an absolute number and percentage or median and range.

## 3. Results

### 3.1. Patients' Characteristics and Distribution of Diagnoses

In 37 procedures, AVPs had been implanted in 36 consecutive patients (56.8% male). The median age was 40 months (range 0.5–504), and the median body weight was 13 kg (range 3.1–61). AVPs were most frequently inserted in SPCA (in 15/37 procedures, 40.6%). Median follow-up duration was 3.25 years (range 0–13) (Table 1).

### 3.2. Overall Procedures

In 37 procedures, 44 AVPs had been implanted in a total number of 36 patients.

The cases are described in detail in Table 2.

We implanted 34 (77.3%) AVP-2 with diameters from 4 to 16 mm and 10 (22.7%) AVP-4 with diameters from 4 to 7 mm. In relation to the diameters of the target lesions, the AVP-2 was oversized by a median of 53% (14–135) and the AVP-4 was oversized by a median of 60% (27–150). Exclusively, AVP-2 was implanted in 28 procedures, exclusively AVP-4 in 5 procedures; 4 patients received both types. The most frequently used plugs were AVP-2 with a diameter of 8 mm (*n*21/44; 47.7%). Nine out of 10 (90.0%) AVP-4 were implanted in SPCA and one (10.0%) in PSA. Primary success in terms of total or subtotal occlusion was achieved in 38/44 (86.3%) implantations. A significant complication occurred in *n*1/44 procedure (2.3%) consisting an obstruction of the left pulmonary artery in a premature infant with a PDA closure (as detailed below).

**Figure 2:**
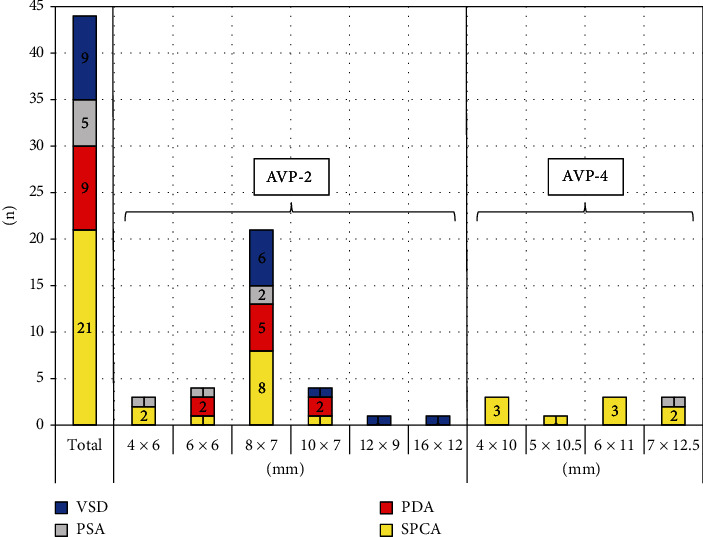
Distribution of plug types and sizes (diameter × unconstrained length in mm). AVP = Amplatzer™ Vascular Plug; PDA = patent ductus arteriosus; PSA = pulmonary sequestration artery; SPCA = systemic-to-pulmonary collateral artery; VSD = ventricular septal defect.

The distribution of plug sizes, types, and treated malformations are specified in Figure 2.

### 3.3. Systemic-to-Pulmonary Arteries

In this group, 10 out of 15 patients (66.7%) had a CHD with univentricular physiology. Four of whom had a partial cavo-pulmonary connection (PCPC) and five of whom had a TCPC; one patient previously received a modified Blalock-Taussig shunt.

In this cohort, 21 AVPs were implanted into 17 vessels; the SPCA originated either from the aorta (*n*5/17), the left subclavian artery (*n*3/17), the right internal mammary artery (*n*6/17), or the left internal mammary artery (*n*3/17). The right internal mammary artery was directly occluded in *n*3/6 and the left in *n*1/3 cases. This procedure was performed if multiple SPCA were derived out of these arteries in a diffuse manner and selective SPCA embolization was not reasonable. As a consequence, the exact number of embolized SPCA cannot be stated.

The following types of AVP were implanted: AVP-2 in *n*12/21 (57.1%) and AVP-4 in *n*9/21 (42.9%). The plugs had diameters of 4, 5, 6, 7, 8, or 10 mm, respectively. The AVPs were oversized by a median of 62% (range 27–150).

**Figure 3:**
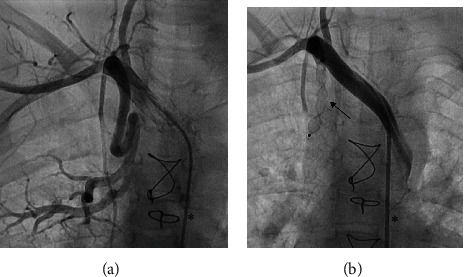
Embolization of a systemic-to-pulmonary artery (SPCA) by AVP-2 (8 mm; arrow) in case 9 (angiography). (a) Large SPCA derived from right subclavian artery. (b) Total occlusion by AVP-2. Endovascular catheters marked by asterisks.

**Figure 4:**
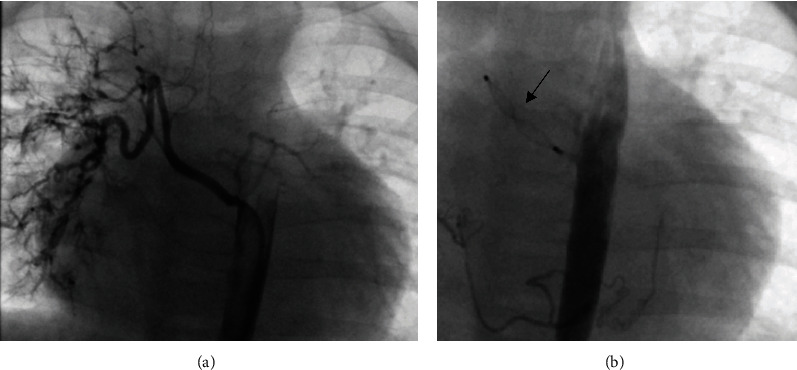
Embolization of a systemic-to-pulmonary artery (SPCA) by AVP-4 (6 mm; arrow) in case 12 (angiography). (a) Large SPCA derived from the descending aorta. (b) Total occlusion by AVP-4.

Primary total or subtotal occlusion of the target vessel was achieved in 17 (81%) procedures. In the remaining four (19%) cases, at least a reduced blood flow was obtained during the procedure. There were no complications associated to the embolization.  Figure 3 shows an SPCA embolization by AVP-2 and Figure 4 the embolization by AVP-4.

### 3.4. PDA

**Figure 5:**
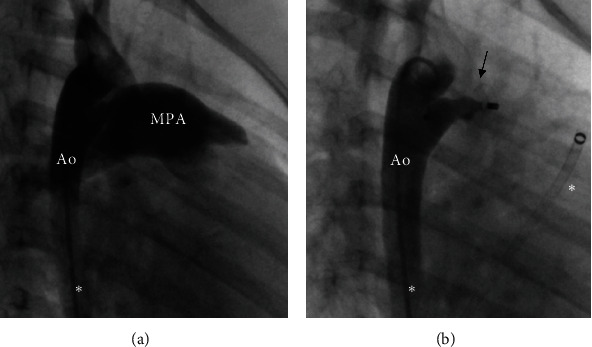
Embolization of a patent ductus arteriosus (PDA) by AVP-2 (10 mm; arrow) in case 23 (angiography). (a) Left-to-right shunt via PDA from aorta (Ao) to main pulmonary artery (MPA). (b) Total occlusion by AVP-2. Endovascular catheters are marked by asterisks.

Occlusion by AVP was performed in nine children with PDA, of which five (55.6%) were preterm infants. AVP-2 was exclusively used; the diameters were 6, 8, and 10 mm, respectively, oversized by a median of 50% (range 20–86).  Figure 5 illustrates a PDA closure.

**Figure 6:**
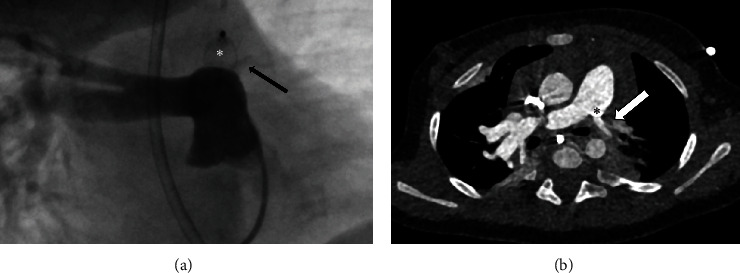
Subtotal obstruction of the left pulmonary artery (arrows) by the ductal AVP-2 (asterisks) 4 months after implantation. (a) Transcatheter angiography. (b) Computed tomography angiography.

Primary PDA closure was achieved in eight (88.9%) patients; one patient had to undergo a second intervention with closure of the residual shunt by an Amplatzer™ Duct Occluder II (18 months later). There was a significant complication in one patient, namely, in a premature infant (body weight 3.1 kg at intervention) with a large PDA that was draining into the left pulmonary artery. An AVP-2 (8 mm) was implanted transarterially. Transvenous catheterization was not possible. Although echocardiographic assessment showed complete PDA closure with only minor obstruction of the left pulmonary artery at the time of discharge, follow-up examination including transcatheter and computed tomography angiography revealed subtotal stenosis 4 months later (Figure 6). Interventional recanalization was not feasible. Hence, removal of the device and PDA closure were performed surgically.

### 3.5. VSD

**Figure 7:**
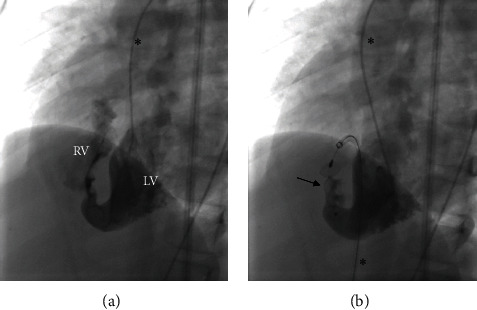
Embolization of a ventricular septal defect (VSD) by AVP-2 (12 mm; arrow) in case 27 (angiography). (a) Left-to-right shunt via VSD (RV = right ventricle; LV = left ventricle). (b) Total occlusion by AVP-2. Endovascular catheters are marked by asterisks.

Nine AVP were implanted in VSD, thereof five (55.6%) muscular VSD and four (44.4%) perimembranous VSD, of which one was a residual VSD after preceding surgical closure. AVP-2 was the only type used in this group. The devices' diameters were 8, 10, 12, and 16 mm with a median oversize of 45% (range 14–100). Total closure was primarily achieved in all procedures without complications. A VSD closure is illustrated in Figure 7.

### 3.6. PSA

**Figure 8:**
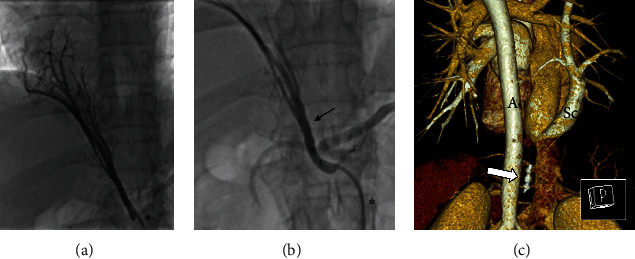
Embolization of a pulmonary sequestration artery (PSA) by AVP-2 (8 mm; arrows) in case 34. (a) PSA derived from the abdominal aorta (angiography). (b) Residual shunt after embolization (angiography). (c) Total PSA occlusion in computed tomography angiography before discharge (Ao = aorta; Sc = scimitar vein). Endovascular catheters are marked by asterisks.

All patients with pulmonary sequestration had an underlying scimitar syndrome. In four patients, five AVPs were implanted: four AVP-2 (diameters 4, 6, and 8 mm, respectively) and one AVP-4 (diameter 7 mm). The median oversize was 53% (range 42–122). Primarily complete closure was achieved in three (75%) patients. In the remaining patient, surgical resection of the sequestration was subsequently performed. Embolization-associated complications did not occur.  Figure 8 illustrates a PSA embolization.

## 4. Discussion

We describe our experiences with transcatheter embolization using AVP in congenital cardiovascular lesions, namely, in SPCA, PDA, VSD, and PSA. Although there are some pre-existing studies dealing with this topic [6, 9, 18], data are still limited.

In 44 AVP embolizations, we achieved a total primary occlusion rate of 86% which is similar to the study of Kubicki et al. with 82% [9].

AVP-2 and AVP-4 have several favorable characteristics: a wide range of diameters from 3 to 22 mm in AVP-2 and 4 to 8 mm in AVP-4, respectively; flexible configuration, therefore suitable for irregularly tortuous or deviated vessels; easy deployment; and low required sheath diameters (4 to 6 French in our cases) that are suitable for a pediatric patient population [18].

AVP-2 has a trilobar configuration. They can adapt to the shape of the target vessel especially in long landing zones. Because the two outer AVP-2 lobes can function as stabilizing discs, this device is also suitable for closure of aneurysmatic perimembranous and muscular VSD, as well as tubular or large elongated PDA [19–21]. The outer discs can be firmly anchored on both sides of the defect, while the middle lobe adjusts in the center of the target. In contrast, AVP-4 is generally not suitable for these procedures because of the absence of the outer discs.

There are different approaches regarding the AVP sizing. The manufacturer's recommendation is oversizing 30–50% in order to ensure occlusion and to avoid migration [4, 22]. Nevertheless, in several published studies, oversizing of 50 to 100% was successfully performed [23–25]. Moreover, the safety and effectiveness of AVP oversizing were previously confirmed also in experimental radial force measurements by Zablah et al. [26]. In our procedures, the median oversize was 53% (14–135) in AVP-2 and 60% (27–150) in AVP-4, respectively, without device migration or vessel rupture.

Transcatheter closure is especially considered in muscular VSD, because their surgical repair is still challenging due to right ventricular trabecularization and therefore difficult exploration of the defects [27]. Aneurysmatic perimembranous VSD are also suitable candidates as the AVP can be placed into the septal bulging. Other devices used for VSD closure are Amplatzer™ duct occluders and genuine VSD occluders, as, for example, the newly developed Lifetech™ Konar-MF or Amplatzer™ VSD occluders [27, 28]. Compared to these devices, the advantage of AVP is their longer middle lobe that can fit in elongated, tunnel-like VSD. Therefore, they can be implanted in a stable position without residual flow. By using AVP-2 in VSD, we achieved a primary total occlusion in 100% without any complications.

In the PDA group, an additional duct occluder had to be implanted in one patient with insufficient closure and significant residual leak. A severe complication occurred in one PDA embolization in terms of subtotal stenosis of the left pulmonary artery caused by the pulmonary disc of the AVP. Surgical repair including AVP removal had to be performed. The AVP was 86% oversized which was still within the range of 50–100% used for PDA closure by Jain et al. [24]. Possible causes for the unfavorable outcome consist in the low body weight and thus small vessels as well as the fact that the duct was atypically merging into the left pulmonary artery. Device-related obstruction of the aorta or the left pulmonary artery is investigated in several published studies. Remarkably, pulmonary stenosis often tends to decrease over time [29, 30]. Nevertheless, size, course, and shape of the targeted PDA have to be approached exactly as well considering the Krichenko duct classification [31, 32]. Short or tortuous PDA should rather be occluded using either genuine duct occluders or coils to avoid excessive protruding into the aorta or the pulmonary trunk. After device implantation, aorta and pulmonary arteries should be evaluated by angiography in order to detect possible obstructions.

Treatment of SPCA is controversially discussed. On one hand, flow and consecutive volume overload via the SPCA are difficult to measure exactly [33]. On the other hand, the presence of collateral arteries is proven to worsen the postoperative outcome after TCPC. It is disputed, whether singular large SPCA or a network of small collaterals have a greater impact [34]. Collateral embolization is usually recommended [35] which is also our approach. Two-thirds of the SPCA patients in our cohort had a univentricular heart. In our opinion, closure of collateral arteries is inevitable to optimize passive pulmonary perfusion in PCPC and TCPC and thus avoid the failure of the Fontan circulation.

For SPCA embolization, we used both AVP-2 (57.1%) and AVP-4 (42.9%). The latter is particularly suitable in this indication because SPCA often have a tortuous or twisted course. For SPCA featuring small diameters or short landing zones, embolization using detachable coils has been practiced for a long time as well [11, 36].

In the SPCA group, there was the highest proportion of only partially occluded targets (19%). It has previously been outlined that the occlusion time by clotting is incalculable in AVP embolization [4, 22]. Considering that the embolizing effect is induced by forming a blood clot within the AVP meshwork, total obstruction may not occur immediately. Administration of heparin during the procedure may further promote delayed clotting. In cases of incomplete occlusion, the implantation of additional devices (e.g., coils) should be taken into consideration.

Finally, we performed PSA embolization by AVP in four patients. On this topic, there are several published case reports and series, for example, Berthod et al., Leoncini et al., or Örün et al. [37–39]. Transcatheter embolization of the PSA can either be the definite treatment by inducing the involution of the sequestration or be performed in preparation of surgical excision to avoid perioperative hemorrhage [17, 40, 41]. PSA usually offers long landing zones and a straight course where uncomplicated implantation of AVP is feasible. Detachable coils may be used for PSA embolization as well, especially in younger children [42]; other approaches are liquid embolization with polyvinyl alcohol, or hybrid operation [43]. In our population, one out of four patients had to undergo a subsequent surgery due to persistent perfusion and recurrent pneumonia.

The main limitations of our institutional study are the limited number of patients and procedures as well as the heterogeneity of the diagnoses included which both can be ascribed to the low incidence of the malformations to be embolized by vascular plugs. Due to their heterogeneity, the diagnostic groups cannot be compared with each other, but the procedures and results within the respective group.

## 5. Conclusion

AVP-2 and AVP-4 are suitable for closure of various congenital cardiovascular malformations with a high primary success rate associated with only few complications. It should be considered in treatment of abnormal vessels as well as selected types of VSD.

## Figures and Tables

**Table 1:** Patients' characteristics and diagnoses.

**Variable**	**All procedures** **(** **n** = 37**)**^†^	**SPCA (** **n** = 15**)**^§^ **(40.6%)**	**PDA (** **n** = 9**)** **(24.3%)**	**VSD (** **n** = 9**)** **(24.3%)**	**PSA (** **n** = 4**)** **(10.8%)**
Age at procedure (months)	40 (0.5–504)	46 (5–336)	13 (3–108)	120 (18–210)	65 (0.5–504)
Female, *n* (%)	16 (43.2)	6 (40)	4 (44.4)	3 (33.3)	3 (75)
Male, *n* (%)	21 (56.8)	9 (60)	5 (55.6)	6 (66.7)	1 (25)
Body weight (kg)	13 (3.1–61)	13 (5.5–50)	6.7 (3.1–25.1)	30 (9.2–61)	16.1 (3.7–56)
Follow-up duration (years)	3.25 (0–13)	6.45 (0–13)	1.5 (0.08–7)	3.5 (1–10)	2.9 (0.2–10)

*Note:* Data are illustrated as absolute numbers and percentage, respectively, median and range.

Abbreviations: PDA = patent ductus arteriosus; PSA = pulmonary sequestration artery; SPCA = systemic-to-pulmonary collateral artery; VSD = ventricular septal defect.

^†^In *n* = 36 patients.

^§^In *n* = 14 patients.

**Table 2:** Case-based description of underlying diseases, target lesions, implantation data, results, and complications.

**Case**	**Sex**	**Age (y)**	**Weight (kg)**	**Underlying disease**	**Univentricular heart**	**Cavo-pulmonary connection (PCPC/TCPC)**	**Target lesion**	**Number of implanted AVP (** **n** **)**	**AVP type**	**AVP diameter (mm)**	**Target diameter (mm)**	**AVP oversizing (%)**	**Primary (sub-)total occlusion**	**Complication**
1	m	0.67	5.5	HLHS	Yes	PCPC	SPCA	1	2	8	5.1	56	Yes	No
2	f	1.92	12	PA, VSD	No	No	SPCA	2	2 2	4 6	2.6 3.4	53 76	Yes Yes	No
3	m	0.42	8	Prematurity	No	No	SPCA	1	4	4	2.8	42	Yes	No
4	f	28.0	50	DORV, TGA	Yes	TCPC	SPCA	1	4	7	4.3	62	Yes	No
5	f	13.0	31	DILV, TGA	Yes	TCPC	SPCA	1	2	8	5.9	35	Yes	No
6	f	11.42	32	PA, VSD	No	No	SPCA	2	2 4	8 7	4.8 4.8	66 45	No Yes	No
7	m	3.33	14	Unbalanced AVSD	Yes	PCPC	SPCA	2	2 4	8 4	4.0 2.5	50 60	Yes No	No
8	m	20.0	47	DILV, TGA	Yes	TCPC	SPCA	1	2	8	3.9	105	Yes	No
9	f	3.33	13	PA, VSD	No	No	SPCA	1	2	8	4.1	95	Yes	No
10	m	4.67	17	HLHS	Yes	TCPC	SPCA	1	2	4	2.8	42	Yes	No
11	m	17.0	49	PA	Yes	TCPC	SPCA	1	2	8	3.4	135	Yes	No
12	m	1.25	8	HLHS	Yes	No	SPCA	1	4	6	4.1	42	Yes	No
13	m	1.0	9	d-TGA	No	No	SPCA	1	4	6	2.4	150	Yes	No
14	m	4.33	13	HLHS	Yes	PCPC	SPCA	3	2 2 4	8 10 4	3.9 5.0 2.9	105 100 27	Yes Yes No	No
15	f	3.83	13	DORV	Yes	PCPC	SPCA	2	4 4	5 6	2.8 3.2	78 87	No Yes	No
16	f	0.83	6	Prematurity	No	No	PDA	1	2	8	5.2	53	Yes	No
17	f	0.5	6	Prematurity	No	No	PDA	1	2	6	4.8	25	Yes	No
18	m	0.58	6	Prematurity	No	No	PDA	1	2	8	4.7	70	Yes	No
19	m	1.25	7.5	No	No	No	PDA	1	2	6	4.0	50	Yes	No
20	f	0.25	3.1	Prematurity	No	No	PDA	1	2	8	4.3	86	Yes	Yes^†^
21	m	1.67	10.5	No	No	No	PDA	1	2	8	5.6	42	Yes	No
22	m	1.08	10.6	Prematurity; PAH	No	No	PDA	1	2	8	5.7	40	Yes	No
23	f	3.0	13	No	No	No	PDA	1	2	10	6.0	66	Yes	No
24	m	9.0	25	No	No	No	PDA	1	2	10	8.3	20	No^§^	No
25	f	10.0	24.5	No	No	No	pVSD	1	2	8	4	100	Yes	No
26	m	2.5	10	Ebstein anomaly, LV noncompaction	No	No	mVSD	1	2	8	5.5	45	Yes	No
27	m	7.67	30	No	No	No	mVSD	1	2	12	8.9	34	Yes	No
28	m	14.92	51	No	No	No	pVSD	1	2	8	7.0	14	Yes	No
29	m	1.5	9	DORV, TGA, Nikaidoh procedure	No	No	mVSD	1	2	8	5.4	48	Yes	No
30	f	10.0	52	Multiple mVSD	No	No	mVSD	1	2	16	9.1	75	Yes	No
31	m	11.25	32	No	No	No	pVSD	1	2	8	7.0	14	Yes	No
32	f	3.75	16	No	No	No	mVSD	1	2	8	5.0	60	Yes	No
33	m	17.5	61	Residual VSD after surgical closure	No	No	pVSD	1	2	10	8.4	19	Yes	No
34	f	42.0	56	Scimitar syndrome, dextrocardia	No	No	PSA	1	2	8	3.6	122	Yes	No
35	f	10.25	26	Scimitar syndrome	No	No	PSA	2	2 4	4 7	n.a.	n.a.	No^¥^	No
36	m	0.04	3.7	Scimitar syndrome	No	No	PSA	1	2	6	3.9	53	Yes	No
37	f	0.42	6	Scimitar syndrome	No	No	PSA	1	2	8	5.6	42	Yes	No

Abbreviations: AVP = Amplatzer™ Vascular Plug; AVSD = atrioventricular septal defect; DILV = double inlet left ventricle; DORV = double outlet right ventricle; HLHS = hypoplastic left heart syndrome; LV = left ventricle; mVSD = muscular ventricular septal defect; n.a. = not available; PA = pulmonary atresia; PAH = pulmonary arterial hypertension; PCPC= partial cavo-pulmonary connection; PDA = patent ductus arteriosus; PSA = pulmonary sequestration artery; pVSD = perimembranous ventricular septal defect; SPCA = systemic-to-pulmonary collateral artery; TCPC = total cavo-pulmonary connection; TGA = transposition of the great arteries.

^†^Stenosis of the left pulmonary artery, subsequent surgery.

^§^Additional occluder implanted for residual defect.

^¥^Subsequent surgical resection.

## Data Availability

The data underlying the present study is available upon reasonable request (corresponding author).

## Abbreviations

AVP: Amplatzer™ Vascular Plug
CHD: Congenital heart defect
PCPC: Partial cavo-pulmonary connection
PDA: Patent ductus arteriosus
PSA: Pulmonary sequestration artery
SPCA: Systemic-to-pulmonary collateral artery
TCPC: Total cavo-pulmonary connection
VSD: Ventricular septal defect
